# Short-term effects of percutaneous electrical nerve stimulation on pain and muscle function in patients undergoing anterior cruciate ligament surgery: a randomized clinical trial

**DOI:** 10.3389/fresc.2025.1501703

**Published:** 2025-04-29

**Authors:** Julio Caballero-López, Marcos Navarro-Santana, Jaime Almazán-Polo, Fernando García-Sanz, María José Díaz-Arribas, Francisco Minaya-Muñoz, Carlos Romero-Morales

**Affiliations:** ^1^ Deparment of Physiotherapy, Faculty of Medicine, Health and Sport, European University of Madrid, Madrid, Spain; ^2^ Clinica CEMTRO, Madrid, Spain; ^3^ Department of Radiology, Rehabilitation and Physiotherapy, Faculty of Nursery, Physiotherapy and Podiatry, Universidad Complutense de Madrid (UCM), Madrid, Spain; ^4^ Grupo InPhysio, Instituto de Investigación Sanitaria del Hospital Clínico San Carlos (IdISSC), Madrid, Spain; ^5^ MVClinic Institute, Madrid, Spain

**Keywords:** ACL rehabilitation, precutaneous electrical nerve stimulation, US guided intervention, ACL surgery, ACL function, femoral nerve

## Abstract

**Introduction:**

Anterior cruciate ligament (ACL) reconstruction surgery is associated with the presence of anterior knee pain and knee extension weakness. Percutaneous electrical nerve stimulation (PENS) is a minimally invasive technique with the objective of neuromodulating the symptoms derived from the intervention. The objective of the study is to analyze the short-term effect of the use of the PENS technique in patients undergoing ACL surgery.

**Materials and Methods:**

A randomized clinical trial was carried out at the CEMTRO clinic in Madrid with 70 participants (*N* = 70) where the effect of the PES intervention in combination with a rehabilitation program (*n* = 35) was compared against a control group of rehabilitation (*n* = 35). The study analyzed changes in pain intensity, pressure pain threshold (PPT) of the vastus medialis, vastus lateralis, quadriceps and patellar tendons, isometric knee extension strength and range of motion of the knee.

**Results:**

Differences were determined in the PENS group compared to the rehabilitation group immediately after the first intervention in the reduction of pain intensity through the VAS scale and in knee extension isometric strength (*p* < 0.05). Both groups showed differences after 12 weeks in the range of motion of the knee in knee flexion and extension, as well as in the PPT of the patellar tendon.

**Conclusion:**

The PENS intervention combined with a rehabilitation program compared to an isolated rehabilitation program showed a short-term reduction in pain intensity and an increase in isometric strength in knee extension in patients undergoing ACL surgery.

**Clinical Trial Registration:**

[ClinicalTrials.gov], identifier [NCT05606250].

## 1 Introduction

Anterior cruciate ligament (ACL) rupture is a common injury typically resulting from acute trauma and is often accompanied by a painful, swollen knee. It is frequently associated with secondary issues such as joint instability, meniscal and cartilage damage, and an increased risk of developing osteoarthritis (1, 2). The incidence is estimated to be 49–75 cases per 100,000 person-years, imposing both socioeconomic and individual burdens (3, 4). Current consensus among orthopedic surgeons recommends that individuals engaged in athletic activities or those with high functional demands on their knees should be offered the option of ACL reconstruction surgery (5, 6). Surgical ACL reconstruction has been associated with improved function, reduced symptoms, and enhanced quality of life compared to individuals who do not undergo surgery (7). However, several studies have reported a decline in quadriceps femoris muscle strength and function following ACL reconstruction, often accompanied by pain, which contributes to delayed recovery of knee joint function (8–10). In this context, the prevalence of anterior knee pain has been estimated to range from 5%–19%, frequently linked to an inability to achieve full knee extension during the early postoperative period. This limitation is associated with quadriceps weakness and alterations in knee and lower limb biomechanics (11).

Percutaneous electrical nerve stimulation (PENS) is a minimally invasive, ultrasound-guided technique that involves delivering electrical current through a solid filament needle. Ultrasound guidance ensures procedural safety and minimizes the risk of adverse events associated with needling techniques applied to sensitive anatomical structures (12). The primary objective of this technique is to achieve both sensory and motor stimulation of peripheral nerves, with specific therapeutic goals tailored according to clinical reasoning based on patient findings and symptomatology (13). The electrical current applied in PENS is biphasic, with frequency ranges from 2–5 Hz to 80–100 Hz and pulse durations varying from 100–450 ms, depending on the desired effect, stimulus intensity, and patient tolerance. This technique delivers electrical stimulation through a needle, typically using acupuncture needles. Although evidence supporting greater pain intensity reduction with PENS is of low quality and the difference is not clinically significant (14), a recent review suggests that PENS provides moderate evidence for pain relief and the reduction of pain-related disability in musculoskeletal conditions (13).

Regarding the associated pain and loss of function after an ACL reconstruction, numerous authors have focused their interventions on targeting the femoral nerve to aid in postoperative pain control and facilitate the immediate restoration of quadriceps function following surger (10, 15–17). The aim of the present study was to evaluate the effects of a PENS of the femoral nerve added into a rehabilitation program in patients with an ACL reconstruction immediately after the surgical procedure on pain, musculoskeletal pain pressure threshold (PPT) alterations, the average (QMeIC) and maximum (QMIC) quadriceps strength during isometric and the knee range of motion (ROM). It was hypothesized that in an early postoperative stage an intervention with a femoral nerve PENS in combination with a rehabilitation program could be more beneficial for the pain intensity, ROM and quadriceps strength with respect to an isolated rehabilitation program in patients with an ACL reconstruction.

## 2 Methods

### 2.1 Study design

The present study was a prospective, randomized, controlled clinical trial (registered at ClinicalTrials.gov as NCT05606250) evaluating individuals over a 12-weeks period between November 2022 and June 2023, following the Consolidated Standards of Reporting Trials (CONSORT) guidelines (18).

### 2.2 Participants

**Figure 1:**
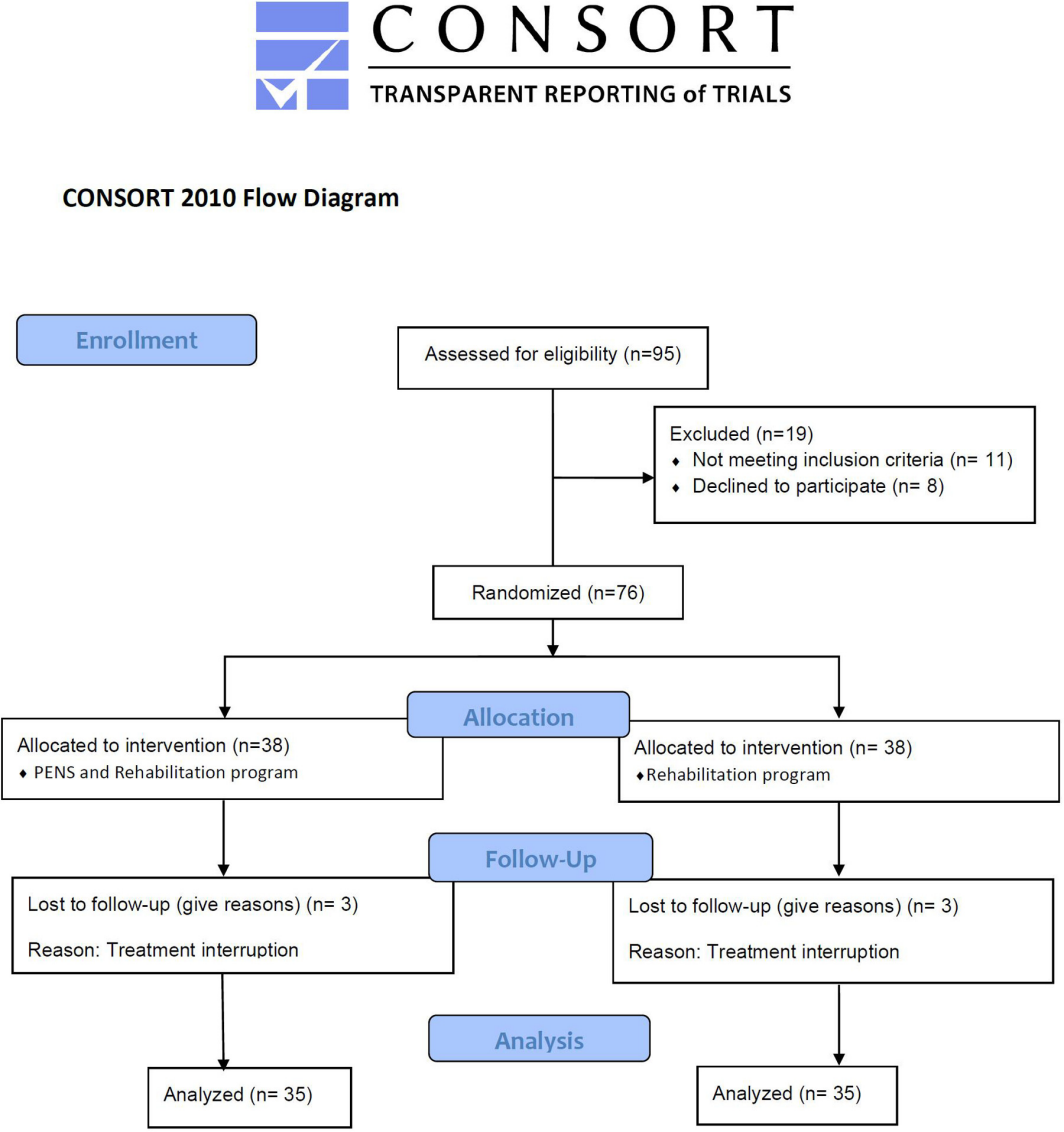
Flowchart of the study according to the CONSORT 2010 statement.

In this study, 70 individuals who undergone ACL surgery were recruited and divided in two groups A and B: group A (*n* = 35) received the femoral nerve PENS plus the rehabilitation program and group B (*n* = 35) who received the isolated rehabilitation program. The selection criteria defined eligible subjects as those who: were aged 18–55 years, individuals who underwent surgical intervention of the ACL within a period of 2–6 weeks post-surgery, had a visual analog scale (VAS) pain score of at least 2 out of 10 points, and have no received any physical therapy. The present pain threshold ensures that the participants presented a clinically relevant level of postoperative pain requiring intervention, while avoiding the inclusion of individuals with minimal or no pain, which could limit the ability to detect treatment-related changes. Exclusion criteria were as follows: metabolic or rheumatic disease, chronic disease, prothesis or osteosynthesis, cardiac disturbances, central nervous system disease, commonly accepted contraindications to invasive techniques, such as epilepsy or belonephobia or fear of needles (Figure 1).

All the participants recruitment and interventions were performed at CEMTRO medical center, FIFA-accredited medical center and supervised thorough the process by a reference medical doctor with more than 25 years of experience in ACL diagnosis and management.

### 2.3 Sample size calculation

Based on prior PENS research, the sample size was calculated using G*Power software, considering pain intensity as the primary outcome. An *a priori* power analysis was conducted using an *F*-test ANOVA for repeated measures to detect between-group differences of 1.5 units, with a standard deviation of 1.75. The estimated sample size required was at least 35 participants per group, accounting for a 15% dropout rate (19).

### 2.4 Randomization

In the research study, the randomization process for dividing participants into two groups was conducted using opaque envelopes. Initially, an equal number of envelopes, each containing a group assignment (either Group A or Group B), were prepared separately for male and female participants to ensure balanced distribution by sex. These envelopes were thoroughly shuffled within each sex category to randomize the order. Participants were then asked to select an envelope at the time of their enrollment in the study, without any prior knowledge of the group they would be assigned to. This selection was done blindly, as the envelopes were opaque and indistinguishable from one another. By employing this method, the study ensured that the allocation of participants to either group was completely random, free from selection bias, and stratified by sex, thereby upholding the integrity and validity of the research outcomes.

### 2.5 Ethical considerations

The study was authorized by the ethics committee of Ntra. Señora del Valme Hospital Universitario (approval code: 0255-N-21) and Universidad Europea Ethics Committee. The study respects the Declaration of Helsinki for human experimentation (20). All the participants signed the informed consent form.

### 2.6 Rehabilitation program for both groups

**Figure 2:**
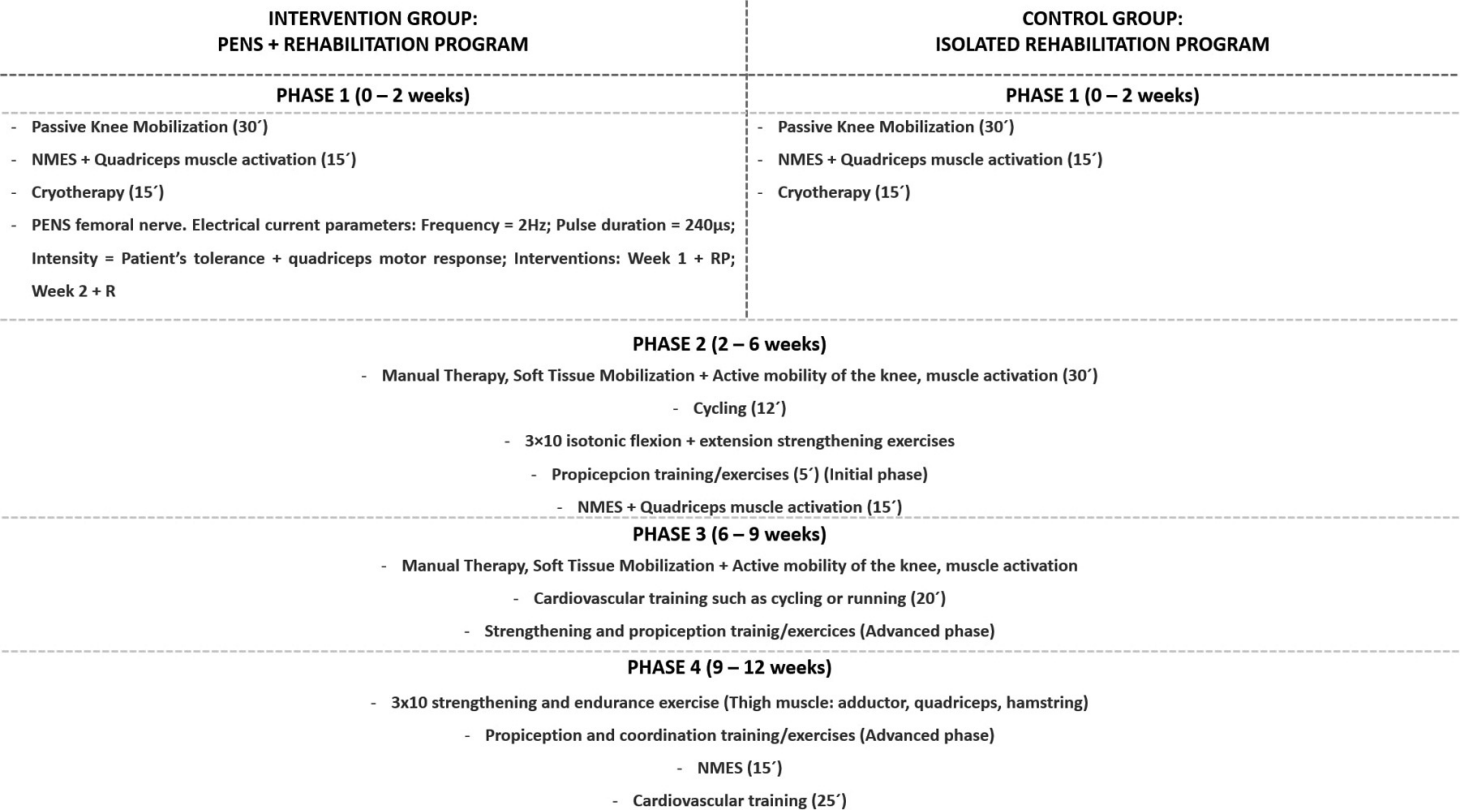
Summary of the study groups: rehabilitation protocols for the intervention (PENS + rehabilitation) and control (isolated rehabilitation) group.

Both groups followed a 12-week rehabilitation program based on clinical practice guidelines for patients after ACL reconstruction adapted to the individual needs and requirements of each patient (21). The program generally consisted of the following phases (Figure 2):

- Phase 1 (Weeks 0–2): 30 min of passive knee mobility (extension and flexion), 15 min of neuromuscular electrical stimulation (NMES) applied to the quadriceps muscle combined with isometric contractions, and 15 min of cryotherapy
- Phase 2 (Weeks 2–6): 30 min of manual therapy, soft tissue mobilization and active knee joint mobility, 12 min of cycling, 3 × 10 repetitions of isotonic flexion and extension strengthening exercises, 5 min of proprioception exercises (Initial pase), 15 min of NMES on the quadriceps muscle, and 10 min of stationary cycling at an intensity based on the participant's tolerance.
- Phase 3 (Weeks 6–9): Manual therapy, soft tissue mobilization and active mobility exercises for the knee and associated periarticular soft tissues, 20 min of cardiovascular training (e.g., running, cycling), and advanced strength and proprioception exercises (Advanced pase).
- Phase 4 (Weeks 9–12): 3 × 10 repetitions of extension, flexion, adductor, and abductor muscle strength and endurance exercises, advanced coordination and proprioception exercises, 15 min of NMES on the quadriceps muscle, and 25 min of cardiovascular training (22).

The rehabilitation program was implemented five times per week for all participants. To ensure the reproducibility of both interventions, the Template for Intervention Description and Replication (TIDieR) checklist was completed and listed according to our registered protocol (23).

### 2.7 PENS intervention group

**Figure 3:**
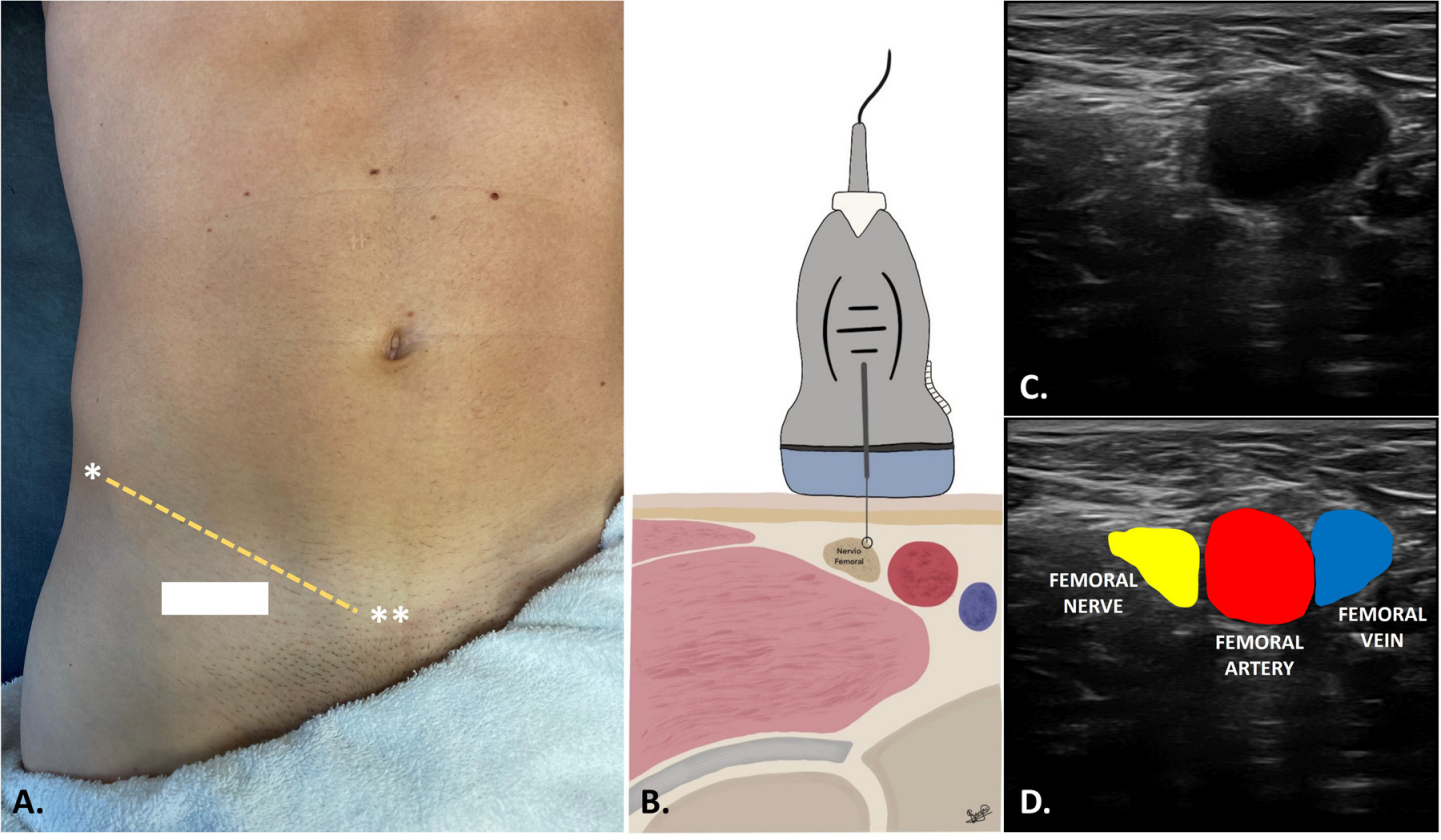
Location of the ultrasound probe and visualization of the femoral nerve for the femoral nerve invasive approach. **(A)** Location of the probe below the inguinal fold at the level of the pubic tubercle (**), below the imaginary line between the anterior superior iliac spine (*); **(B)** Representation of the peripheral placement of the needle in relation to the position of the femoral nerve with respect to the femoral vascular structures **(C,D)**. The needle entry approach was performed “out of plane” by passing the needle over the lateral edges of the perineurium (white dashed arrows showing the needle positioning) at the lateral or superior border of the nerve.

Participants assigned to the experimental group received two sessions of PENS targeting the femoral nerve. The first intervention was administered during the initial session, with data collected before the intervention (Baseline) and immediately afterward (Post-Intervention 1). The second PENS intervention was performed one week later, with data collected at the beginning of the session (Pre-Intervention 2) and immediately after the intervention (Post-Intervention 2). In the current study, we applied the electrical current with a 30 × 0.40 mm needle (Agupunt®) as close as possible to the femoral nerve. The femoral nerve was US—guided with an ultrasound system (Sonoscape E2, Spain) with a linear transducer of 12 MHz to provide the highest accuracy for needle insertion and safety for the patients. The images were collected immediately below the inguinal fold at the level of the pubic tubercle, taking as reference the imaginary line between the anterior superior iliac spine and the pubis to obtain the transverse view (short axis) of the femoral nerve (Figure 3). Once the short axis of the femoral nerve was identified at the level of the femoral triangle within the muscular lacuna, the invasive “out of plane” approach was performed by inserting the needle under ultrasound guidance along the probe's short axis, with an entry angle of 90° relative to the skin. The needle's advancement was continuously monitored to ensure its correct positioning along the upper and lateral peripheral edges of the nerve. PENS procedure was developed with an ITO ES-160 (Ito Co. Ltd., Tokyo, Japan) device, at 2 Hz frequency, with 240 μs pulse duration (24). The electrical current was increased at an intensity of a visible motor response of the femoral nerve innervated musculature (25) (Figure 2).

### 2.8 Outcomes

Pain intensity was assessed with a visual analogue scale (VAS), a valid, responsive, and frequently used which consist in a 10 cm bidirectional straight line with two labels in both edges, “no pain” and “worst possible pain” (26). Subjects were instructed to draw a vertical mark on the line to indicate their pain intensity level. VAS reported an excellent test-retest reliability and a minimum detectable change (MCD) of 0.08 (27).

PPT was assessed from 0–10 kg/cm^2^ with a mechanical algometer (FDK/FDN, Wagner Instruments, Greenwich, CT). The most hyperalgesic area of the vastus medialis and vastus lateralis muscles and quadricipital and patellar tendons were evaluated in supine position. With continuously increasing pressure, the soft tissue targeted was compressed with the metal rod of the algometer. The selected threshold aimed to identify the earliest indication of hyperalgesia in the targeted tissue, reflecting changes in peripheral and central sensitization associated with post-surgical recovery. Participants were instructed to report the exact moment at which they experienced pain. The intra-rater reliability for PPT was excellent (0.93–0.97) with a MCD of 1.53–1.62 (28). This protocol is consistent with previous studies that support the use of PPT as a reliable and sensitive indicator of mechanosensitivity, particularly at the knee. For example, Mutlu et al. reported post-intervention increases in medial knee PPT from 5.47 ± 2.99–7.58 ± 2.97 lb, with a SEM of 0.66, MDC of 1.19, and a moderate-to-large effect size of 0.70. Similarly, Paungmali et al. demonstrated excellent reliability for lumbar PPT measures (ICC = 0.99; SEM = 1.19), further supporting the robustness and clinical utility of single-session PPT assessments in musculoskeletal populations (29).

Isometric muscle force was assessed with the ActivForce (AF; Activbody, San Diego CA) for the knee extension (30). Participants were placed sitting at the end of the table with a belt located on the ankle of the assessed leg with a 90° degrees knee flexion ankle. To assess the maximal isometric contraction of the quadriceps (QMIC) and the mean contraction strenght (QMeIC), participants were instructed to perform a maximal knee extension against the dynamometer for 4 s. Inter-examiner reliability of knee extensors and flexors were 0.97 and 0.95, respectively, with a MDC of 33.09 Nm and 21.45 Nm for knee extensors and flexors, respectively (31). Three repetitions of each measurement were performed to establish the average of the results.

Knee ROM was assessed using a universal goniometer with one-axis joints with two-arms (one movable and one fixed arm). This procedure demonstrates excellent intra-rater reliability, with intraclass correlation coefficients (ICCs) of 0.997 for flexion and 0.98 for extension. Similarly, inter-rater reliability shows ICCs of 0.98 for flexion and 0.92 for extension, respectively (32). All study measurements will be performed in the same temporal sequence for participants in both groups and by the same evaluator, who will be blinded to group allocation. The first evaluation was carried out before the first session (Baseline), the second measurement after the first intervention (Post-Intervention 1), the third measurement before the second intervention session one week later (Pre-Intervention 2) and the fourth measurement after said session (Post—Intervention 2), and finally the fifth and sixth measurement at 4 (4-weeks) and 12 (12-weeks) weeks respectively.

### 2.9 Statistics

The statistical analysis was performed using the Statitstical Package for Social Sciences (SPSS) v.23.0 for Windows (IBM SPSS Statistics, NY: IBM Corp.) and Jamovi v.2.3 (https://www.jamovi.org) was carried out by an independent statistician. An α error of 0.05 (95% confidence interval) and a desired power of 80% (β error of 0.2) were used for all the statistical tests. The Shapiro–Wilk test was used for normality assumption. For the baseline comparison, the Student *t* test was used, considering the homogeneity of variance following Levene's test. A two-way analysis of variance (ANOVA) for repeated measures was employed to examine the intra-subject (Baseline, Post-Intervention 1, Pre-Intervention 2, Post-intervention 2, 4-weeks, 12-weeks) and inter-subject (treatment*group) effects for the dependent variables. The assumption of homoscedasticity was assessed using Levene's test, and when this assumption was violated, Welch's statistical test was applied. The assumption of sphericity was evaluated using Mauchly's test, and when violated, the Greenhouse-Geisser correction was applied for epsilon values >0.75, whereas the Huynh-Feldt correction was used for epsilon values <0.75. Effect sizes were calculated using partial eta squared (*η*p²) coefficients and interpreted according to the following thresholds: 0.01 (small effect size), 0.06 (medium effect size), and 0.14 (large effect size). *post-hoc* analyses were conducted using Bonferroni's correction, adjusting the significance level to *p* < 0.008 to account for the six time-point measurements.

## 3 Results

Sociodemographic data did not statistically differ between groups (*p* > 0.05) (Table 1). Pain intensity, PPT for vastus medialis and lateralis muscles, ROM and maximal isometric contraction (QMIC) reported benefits with respect to the baseline but did not show significant differences (*p* > 0.05) between the intervention groups (Tables 2, 3). *post-hoc* analysis reported for pain intensity significant group differences in favor the PENS treatment immediately posterior the first intervention (Baseline vs. Post-Intervention 1; *p* = 0.00, *η*p^2^ = 0.14) and the week after (Baseline vs. Post-Intervention 2; *p* = 0.003, *η*p^2^ = 0.29), as in the comparison with the pain at 4-weeks (Baseline vs. 4-weeks; *p* = 0.003, *η*p^2^ = 0.33). Regarding the ROM *post-hoc* analyses between the Baseline and 12-weeks assessments, significant differences were found in both groups for the knee flexion (*p* < 0.001) and extension (*p* = 0.021). The PPT of the patellar tendon showed significant differences between Baseline vs. 12-weeks measurements in PENS group with respect to the Control group. For quadriceps during mean isometric contraction (QMeIC), significant differences were found immediately after first intervention comparing PENS group (Baseline vs. Post-Intervention 1) with respect to the control group (*p* = 0.049) (Table 3). Despite this acute effect in strength, no interaction of medium-long term effect between group * time was reported (*F* = 0.16, *p* = 0.695, *η*p^2^ < 0.01).

**Table 1:** Sociodemographic characteristics of the study sample.

Data		PENS (*n* = 35)	Control (*n* = 35)	*P*-value
Age, y		30.6 ± 9.68	30.66 ± 10.79	0.939
Weight, kg		74.26 ± 11.48	71.11 ± 13.51	0.441
Height, m		1.75 ± 0.9	1.71 ± 0.5	0.063
BMI, kg/m^2^		24.17 ± 3.49	24.25 ± 3.77	0.981
Sex	Women	14 (20%)	14 (20%)	1.000
	Men	21 (30%)	21 (30%)	

Frequency and relative percentage (%) as well as Chi-square test was used for was used for differences assessment between sex distribution per groups.

**Table 2:** Dependent variables of the study of pressure pain threshold and pain intensity in the different time phases, with means, standar deviaitonss and IC’s 95% represented.

Measure		PENS	Control	Intrasubject Effects	
		(*n* = 35)	(*n* = 35)	Time value	Treatment X Time
				*F*; *P* (Eta^2^)	*F*; *P* (Eta^2^)
VAS, (0–10)	Baseline	2.34 ± 1.78 (1.73–2.95)^a^^,^^b^^,^^c^	2.51 ± 1.76 (1.91–3.12)	*F* = 12.39	*F* = 1.51
				*P* = **0.001** (0.154)	*P* = 0.216 (0.02)
	Post-Intervention 1	1.60 ± 1.31 (1.15–2.05)	2.60 ± 2.08 (1.89–3.31)		
	Pre-Intervention 2	1.54 ± 1.07 (1.18–1.91)	2.17 ± 1.62 (1.62–2.73)		
	Post-Intervention 2	1.34 ± 1.0 (1.0–1.69)	2.17 ± 1.5 (1.65–2.69)		
	4-weeks	1.17 ± 0.45 (1.02–1.33)	1.80 ± 1.30 (1.35–2.25)		
	12-weeks	1.06 ± 0.42 (0.91–1.20)	1.51 ± 0.95 (1.19–1.84)		
VM-PPT, kg/s	Baseline	7.03 ± 1.88 (6.38–7.68)	6.43 ± 1.94 (5.76–7.10)	*F* = 27.61	*F* = 0.16
				*P* = **0.001** (0.29)	*P* = 0.951 (0.01)
	Post-Intervention 1	7.11 ± 1.88 (6.46–7.75)	6.30 ± 2.01 (5.61–6.99)		
	Pre-Intervention 2	7.42 ± 2.0 (6.73–8.10)	6.68 ± 2.10 (5.96–7.40)		
	Post-Intervention 2	7.58 ± 1.89 (6.93–8.23)	6.79 ± 1.94 (6.13–7.46)		
	4-weeks	8.44 ± 1.81 (7.82–9.06)	7.60 ± 1.94 (6.92–8.28)		
	12-weeks	8.73 ± 1.51 (8.21–9.24)	7.89 ± 1.93 (7.22–8.55)		
VL- PPT, kg/s	Baseline	7.23 ± 1.72 (6.63–7.82)	6.23 ± 1.94 (5.56–6.89)	*F* = 25.89	*F* = 0.84;
				*P* = **0.001** (0.28)	*P* = 0.505 (0.01)
	Post-Intervention 1	7.30 ± 1.99 (6.61–7.98)	6.02 ± 2.27 (5.31–6.87)		
	Pre-Intervention 2	7.08 ± 1.67 (6.51–7.66)	6.46 ± 2.10 (5.73–5.18)		
	Post-Intervention 2	7.52 ± 1.62 (6.96–8.08)	6.53 ± 2.09 (5.82–7.25)		
	4-weeks	8.41 ± 1.52 (7.89–8.93)	7.16 ± 2.13 (6.43–7.89)		
	12-weeks	8.70 ± 1.49 (8.19–9.21)	7.94 ± 1.77 (7.33–8.55)		
Quadricipital tendon PPT, kg/s	Baseline	8.01 ± 1.81 (7.39–8.63)	6.93 ± 2.47 (6.08–7.78)	*F* = 22.69	*F* = 1.07
				*P* = **0.001** (0.25)	*P* = 0.374 (0.02)
	Post-Intervention 1	8.22 ± 1.71 (7.63–8.81)	7.24 ± 2.45 (6.40–8.08)		
	Pre-Intervention 2	8.44 ± 1.87 (7.80–9.08)	7.44 ± 2.24 (6.67–8.21)		
	Post-Intervention 2	8.47 ± 1.84 (7.83–9.10)	7.64 ± 2.24 (6.86–8.41)		
	4-weeks	9.00 ± 1.54 (8.47–9.53)	8.35 ± 1.95 (7.68–9.02)		
	12-weeks	9.37 ± 1.36 (8.90–9.83)	9.03 ± 1.39 (8.56–9.51)		
Patellar tendon PPT, kg/s	Baseline	7.82 ± 2.07 (7.11–8.53)	7.50 ± 2.21 (6.74–8.26)	*F* = 11.39	*F* = 1.03
				*P* = **0.001** (0.14)	*P* = 0.384 (0.01)
	Post-Intervention 1	8.27 ± 2.08 (7.56–8.99)	7.52 ± 2.15 (6.78–8.26)		
	Pre-Intervention 2	8.41 ± 1.94 (7.74–9.07)	7.61 ± 2.02 (6.92–8.30)		
	Post-Intervention 2	8.69 ± 1.95 (8.03–9.36)	7.69 ± 2.08 (6.97–8.40)		
	4-weeks	9.06 ± 1.47 (8.56–9.75)	8.32 ± 1.75 (7.72–8.92)		
	12-weeks	9.35 ± 1.19 (8.94–9.75)	8.25 ± 1.95 (8.94–9.75)		

VAS, visual analogue scale; VM-PPT, vastus medialis pain pressure threshold; VL-PPT; PENS, percuatenous electrical nerve stimulation.

^a^ Time differences from Baseline. vs. Post-Intervention 1 with *p*-value < 0.008.

^b^ Time differences from Baseline vs. Post-Intervention 2 with *p*-value < 0.008.

^c^ Time differences from Baseline. vs. 4-week with *p*-value < 0.008; For all analyses, *P* < .05 (for a confidence interval of 95%) was considered statistically significant (**bold**).

**Table 3:** Dependent variables of the study of range of motion (ROM) and strength in the different time phases, with means, standar deviaitonss and IC’s 95% represented.

Measure		PENS	Control	Intrasubject Effects	
				Time value	Treatment X Time
		(*n* = 35)	(*n* = 35)		
				*F*; *P* (Eta^2^)	*F*; *P* (Eta^2^)
Extension ROM, (°)	Baseline	−1.14 ± 2.82 (−2.11–0.17)	−4.29 ± 5.48 (−6.17–2.40)	*F* = 13.02	*F* = 4.58
				*P* = **0.001** (0.168)	*P* = **0.001** (0.063)
	Post-Intervention 1	−0.46 ± 1.92 (−1.11–0.20)	−2.69 ± 4.03 (−4.07–1.30)		
	Pre-Intervention 2	−0.86 ± 1.96 (−1.53–0.18)	−3.23 ± 4.62 (−4.81–1.64)		
	Post-Intervention 2	−0.57 ± 1.44 (−1.07–0.08)	−2.14 ± 3.40 (−3.31–0.98)		
	4-weeks	−0.26 ± 0.95 (−0.58–0.07)	−1.14 ± 2.78 (−2.10–0.19)		
	12-weeks	0.0 ± 0.0 (0–0)	−0.14 ± 0.85 (−0.43–0.15)		
Flexion ROM, (°)	Baseline	100.8 ± 15.75 (95.45–106.27)	100.9 ± 19.55 (94.23–107.66)	*F* = 168.74	*F* = 2.07
				*P* = **0.001** (0.71)	*P* = 0.121 (0.03)
	Post-Intervention 1	104.6 ± 16.64 (98.88–110.32)	106 ± 17.23 (100.11–111.95)		
	Pre-Intervention 1	110.9 ± 12.28 (106.73–115.16)	108.1 ± 14.01 (103.30–112.32)		
	Post-Intervention 2	113.6 ± 12.47 (109.32–117.88)	113.6 ± 13.89 (108.83–118.37)		
	4-weeks	128.9 ± 8.90 (125.86–131.97)	125.8 ± 10.7 (122.13–129.53)		
	12-weeks	135.0 ± 6.73 (132.74–137.37)	136.51 ± 6.3 (134.33–138.70)		
Q-MIC, (Nw)	Baseline	149.1 ± 80.2 (121.54–176.66)	149.6 ± 81.1 (121.78–177.52)	*F* = 132.25	*F* = 0.75
				*P* = **0.001** (0.66)	*P* = 0.511 (0.01)
	Post-Intervention 1	173.6 ± 71.7 (149.10–198.24)	161.5 ± 91.99 (129.97–193.17)		
	Pre-Intervention 2	182.9 ± 78.6 (155.94–209.95)	185.4 ± 95.73 (152.57–218.34)		
	Post-Intervention 2	211.2 ± 79.1 (184.02–238.38)	203.1 ± 89.47 (172.44–233.91)		
	4-weeks	251.9 ± 84.77 (222.86–281.11)	233.99 ± 89.26 (203.32–264.65)		
	12-weeks	315.4 ± 82.98 (286.97–343.98)	298.5 ± 108.5 (261.28–335.86)		
Q-MeIC, (Nw)	Baseline	100.1 ± 55.38 (81.17–119.21)	104.5 ± 61.58 (83.35–125.66)	*F* = 123.78	*F* = 0.16
				*P* = **0.001** (0.65)	*P* = 0.695 (0.01)
	Post-Intervention	123.6 ± 52.65 (105.53–141.70)	116.3 ± 71.21 (91.91–140.83)		
	Pre-Intervention 2	138.2 ± 66.08 (115.54–160.94)	136 ± 78.55 (109.11–163.07)		
	Post-Intervention 2	154 ± 64.64 (131.81–176.22)	153.6 ± 74.72 (128.01–179.35)		
	4-weeks	188.7 ± 72.97 (163.70–213.83)	175.64 ± 77.55 (149.01–202.28)		
	12-weeks	232.5 ± 65.88 (209.87–255.13)	215.6 ± 86.37 (185.95–245.29)		

Q-MIC, quadriceps maximal isometric contraction; Q-MeIC, quadriceps mean isometric contraction; PENS, percutaneous electrical nerve stimulation.

Bold values denote significant differences were determined, *P*-value <0.05.

## 4 Discussion

To the best of our knowledge, this is the first randomized clinical trial to evaluate the effects of ultrasound-guided percutaneous electrical nerve stimulation (PENS) in patients undergoing ACL reconstruction. The main finding of this study indicates that the PENS group demonstrated significant short-term improvements in perceived pain intensity and active knee extension isometric strength following the first intervention, compared to the control group. Nevertheless, both groups exhibited beneficial effects on PPT, ROM, pain intensity, and knee extension strength (QMeIC and QMIC) at the 12-week follow-up. These results suggest a potential short-term advantage of PENS in reducing pain and enhancing quadriceps strength immediately after the intervention.

### 4.1 PENS outcomes on pain variables

The reduction in pain observed with the combination of PENS and a rehabilitation program, compared to rehabilitation alone, is an important finding in the early postoperative management of pain. However, despite these initial improvements, the comparison of long-term effects (12 weeks) across repeated measures (six assessments) showed no significant differences between groups. This suggests that while PENS may help modulate pain symptoms in the short term, its effects may diminish over time, ultimately leading to similar outcomes as rehabilitation alone. Although the present study provides evidence of short-term effects of PENS, the current follow-up period of 12-week may be insufficient to detect long-term benefits or potential sustained neuromodulatory effects on pain and muscle function. Chronic post-surgical pain and long-standing quadriceps inhibition remain significant concerns in ACL rehabilitation. Therefore, future studies should explore whether repeated or maintenance PENS sessions over a longer period, possibly in the later stages of rehabilitation, can help reduce chronic pain development and enhance long-term muscle function recovery. Additionally, assessing outcomes beyond 12-week would help clarify whether the neuromodulatory mechanisms triggered by PENS persist or require continued stimulation.

Fernández de las Peñas et al. reported findings consistent with those of our study, demonstrating short-term improvements in pain, function, and pain intensity. Similar effects were observed in the medium and long term in patients undergoing carpal tunnel surgery compared to the PENS group (19). However, it is important to note that short-term differences in their study were classified within the first three months, whereas long-term follow-up extended up to 12 months. Additionally, some evidence supports the effectiveness of PENS in managing chronic pain conditions, including chronic low back pain, knee and ankle pain, and certain neuropathic pain syndromes (13, 19). Regarding its impact on pain intensity and disability, Plaza-Manzano et al. conducted a meta-analysis indicating that PENS may reduce pain intensity, though its effects on disability in musculoskeletal disorders appear limited (33).

The immediate clinical improvement of symptoms may represent the opening of a therapeutic window for both pain management and the modulation of inhibition phenomena observed in post-surgical processes. The biological mechanisms underlying these immediate effects may involve the blockade of nociceptive input through the regulation of neuroinflammation and neurogenic excitability, which are characteristic of the early stages following surgery (13). Although chronic pain involves processes beyond inflammation and neuroinflammation, stages of chronic neuroinflammation have been observed in patients with chronic pain, such as in the case of fibromyalgia (34). The presence of various neuroinflammatory mediators during post-surgical stages—along with altered sensory pain modulation and glial cell activation—has been proposed as part of the pathophysiology of what is defined as “post-surgical pain.” In this context, non-pharmacological interventions such as PENS or neuromodulation techniques, including spinal cord stimulation, have been suggested as therapeutic strategies capable of modulating pain and reducing post-surgical neuroinflammation (35). Furthermore, other therapeutic approaches, such as neurodynamic mobilization—which aims to modulate the nervous system through mechanical stimuli and movement—have demonstrated a reduction in pain and an improvement in grip strength following the mobilization of the median nerve in populations with osteoarthritis of the carpometacarpal joint (36). In this context, combining therapeutic strategies that focus on modulating centrally mediated pain through the stimulation of peripheral neural tissue represents a promising avenue for future research in the management of post-surgical pain (37, 38).

### 4.2 PENS outcomes in functional variables, strength and range of motion

Regarding the effect of PENS on improving knee extension strength immediately, previous research has documented similar results on muscle function (39, 40). The effects of PENS depend on both the needle's placement, determined by the topographic distribution of motor and somatic axons within the nerve trunk, and the modulation of electrical parameters. These parameters include pulse width, insertion site, frequency, and current intensity, among others. By adjusting these factors, the motor response can be tailored, offering potential advantages over techniques such as neuromuscular electrical stimulation (NMES), as PENS activates motor units along the entire length of the nerve rather than only superficial motor units (41–43).

Therefore, addressing quadriceps activation failure resulting from neural inhibition—specifically identified as iatrogenic inhibition following ACL intervention—should be considered a key therapeutic target in this population. The limited evidence supporting the effectiveness of therapies such as transcutaneous electrical nerve stimulation (TENS) or NMES in managing neural inhibition, especially when compared to other interventions like cryotherapy or exercise, has been noted in the literatura (44). Moreover, direct stimulation of the nerve trunk appears to provide a more effective means for restoring resting motor potentials, modulating the sensitivity of articular receptors, and influencing spinal and cortical excitability. However, further research is needed to determine the effects of different current parameters applied through the PENS technique, with the aim of optimizing motor responses to modulate iatrogenic inhibition in patients undergoing ACL reconstruction. Beyond its analgesic effects, PENS also demonstrated a clinically relevant capacity to enhance quadriceps activation in the immediate postoperative phase. This dual impact—on both pain and neuromuscular performance—positions PENS as a valuable adjunct for promoting early functional recovery through simultaneous modulation of nociceptive input and facilitation of motor output.

### 4.3 Clinical relevance

The clinical relevance of these findings lies in the potential for PENS to be used as a complementary strategy during the early stages of rehabilitation following ACL reconstruction. The immediate reduction in pain and the enhancement of quadriceps activation observed in this study suggest that PENS may serve as an effective tool to facilitate early neuromuscular recovery and improve patient engagement with the rehabilitation process. Its integration into standard care protocols could help overcome initial barriers to movement and muscle recruitment, promoting faster progression through rehabilitation phases. Moreover, the rapid onset of benefits positions PENS as a valuable option for clinicians aiming to optimize early functional outcomes and reduce the impact of postoperative pain and quadriceps inhibition in the short term.

### 4.4 Strength and limitations

The following strengths and limitations should be considered in the results of this study. The findings of the present study provide immediate effects on pain reduction and strength recovery; thus, PENS may offer significant advantages for managing post-surgical pain and addressing long-term neuromuscular deficits. Previous studies have shown the effectiveness of PENS in conditions such as carpal tunnel syndrome and chronic low back pain, which demonstrated sustained improvements in pain intensity and functional outcomes over extended periods (19, 33). These findings suggest that PENS could play a crucial role in mitigating chronic neuroinflammation and enhancing motor function in patients with persistent iatrogenic quadriceps inhibition following ACL surgery. The ability of PENS to modulate neuroinflammatory processes and restore motor unit activation through targeted stimulation of the femoral nerve makes it a promising adjunct to traditional rehabilitation protocols. In this line, this approach could prevent chronic pain syndromes and optimize neuromuscular recovery in this population. Future studies should investigate the integration of PENS into later phases of rehabilitation, with extended treatment durations and additional nerve targets, such as the saphenous and obturator nerves, to maximize therapeutic efficacy.

Moreover, certain limitations must be considered in research design. Firstly, the limitation of the short-term effect of the PENS intervention compared to the control group could call into question the real benefit between conservative management and the application of an invasive approach. In this sense, a possible explanation for the lack of differences observed in the medium and long term could be the limited number of PENS interventions, as only two sessions were performed in the intervention group—one in the first week and another in the following week after surgery. This may have been insufficient to produce sustained effects over time. Therefore, further studies are needed to evaluate the impact of PENS in later phases of the rehabilitation process, with a greater number of applications over an extended period. Secondly, the role of different psychological yellow flags in the evolution of patients, such as fear-avoidance behaviors, anxiety, or psychological profile, could play an interesting role in long-term evolution was not considered. Thirdly, the lack of evidence regarding the optimal dosing strategy for PENS therapy must be considered. Future research comparing different treatment doses (sessions per week) and stimulation parameters (frequency, pulse width, application duration, and achieved response) is needed to establish more standardized protocols and better determine the most effective therapeutic approach. Lastly, targeting additional neural structures involved in the sensory innervation of the knee and motor function of the thigh—such as the posterior branch of the obturator nerve, the saphenous nerve, the tibial nerve, the genicular branches, as well as the common fibular nerve and its articular branch—should be considered in future research to enhance the effectiveness of the PENS technique.

## 5 Conclusion

The minimally invasive approach to PENS, combined with a conservative rehabilitation program in patients undergoing ACL surgery, demonstrated immediate improvements in pain intensity reduction and knee extension isometric strength compared to the isolated rehabilitation program. However, both groups showed improvements in pain, ROM, and strength over time, with no significant long-term differences between groups, except for knee extension ROM. Future studies with longer intervention durations are needed to further explore the effects of the PENS technique on muscle function and pain management in post-surgical patients over the medium and long term.

## Data Availability

The datasets presented in this article are not readily available because Legal restrictions due to data confidentiality. Requests to access the datasets should be directed to Jaime Almazan Polo; aime.almazanpolo@gmail.com.
